# Primary hypertension is a disease of premature vascular aging associated with neuro-immuno-metabolic abnormalities

**DOI:** 10.1007/s00467-015-3065-y

**Published:** 2015-02-28

**Authors:** Mieczysław Litwin, Janusz Feber, Anna Niemirska, Jacek Michałkiewicz

**Affiliations:** 1 Department of Nephrology and Arterial Hypertension, The Children’s Memorial Health Institute, Warsaw, Poland; 2 Department of Pediatrics, Children’s Hospital of Eastern Ontario, University of Ottawa, Ottawa, Canada; 3 Department of Microbiology and Immunology, The Children’s Memorial Health Institute, Warsaw, Poland; 4 Department of Immunology, Medical University, Bydgoszcz, Poland

**Keywords:** Primary hypertension, Children, Immunologic activity, Sympathetic activity, Metabolic syndrome, Target organ damage, Vascular aging

## Abstract

There is an increasing amount of data indicating that primary hypertension (PH) is not only a hemodynamic phenomenon but also a complex syndrome involving abnormal fat tissue distribution, over-activity of the sympathetic nervous system (SNS), metabolic abnormalities, and activation of the immune system. In children, PH usually presents with a typical phenotype of disturbed body composition, accelerated biological maturity, and subtle immunological and metabolic abnormalities. This stage of the disease is potentially reversible. However, long-lasting over-activity of the SNS and immuno-metabolic alterations usually lead to an irreversible stage of cardiovascular disease. We describe an intermediate phenotype of children with PH, showing that PH is associated with accelerated development, i.e., early premature aging of the immune, metabolic, and vascular systems. The associations and determinants of hypertensive organ damage, the principles of treatment, and the possibility of rejuvenation of the cardiovascular system are discussed.

## Introduction

In the 1940s, Irvin Page developed his mosaic theory of the pathogenesis of primary hypertension (PH). The basis of this concept relied on the recognition of many interconnected factors, including all known mechanisms playing a role in the elevation of blood pressure (BP) [1]. The so-called “Page’s Mosaic” described an interplay between anatomic, genetic, metabolic, nervous, hemodynamic, and environmental factors. The elevation of BP and the eventual development of PH were the end result of the interplay of all these factors, indicating that the pathogenesis of PH is multifactorial. Despite the fact that many new mechanisms and factors influencing BP control have been discovered since then, the mosaic theory is still valid [2].

Another theory was described over 40 years ago by Arthur Guyton. His model of BP regulation is based on the relationship between volume and peripheral vascular resistance. It explains the main mechanisms in regulating peripheral blood flow, along with adaptations of other neural, hormonal, and autacoid systems concerning the changes in resistance–volume relations. Moreover, it describes not only physiological regulations of BP and peripheral flow but also explains the pathogenesis of most of the secondary forms of arterial hypertension. The main mechanisms of BP control described by Guyton also work for PH, however both models fail to explain the inciting events in PH. Recent findings from experimental and clinical studies have suggested that metabolic abnormalities, sympathetic nervous system (SNS) alterations, immune activation, early arterial changes and their interrelationships may in fact represent inciting events in the development of sustained PH.

Children with PH in the early stages of hypertensive disease are usually not affected by other confounding factors typically present in adulthood; issues such as diabetes mellitus, nicotinism, and atherosclerosis. Thus, data from pediatric studies may shed more light on the pathogenesis of early stages of PH.

## Intermediate phenotype of PH in childhood

### Anthropometrical and metabolic abnormalities

The relationship between body mass index (BMI) and BP explains why an increasing prevalence of obesity is associated with an increased prevalence of PH among children and adolescents [3, 4]. It was reported a decade ago that a typical phenotype in a child in New York with PH was obesity [5]. The authors also noted that there was a steady increase in the prevalence of obesity and being overweight by observing an increasing severity of hypertension from borderline hypertension to sustained hypertension. This was confirmed by studies from Europe in hypertensive adolescent subjects who were taller and heavier than their normotensive peers do [6]. The association between BMI and the associated metabolic abnormalities (such as hyper-insulinism, insulin resistance, IR) and BP levels is almost linear; such a relationship is observed as early as 4 years old [7]. Moreover, Sinaiko et al. found that elevated insulin concentrations and a lower glucose uptake at the age of 13 preceded the increase of systolic BP at the age of 19 [8]. The data indicate that this is not related to BMI but rather an increased ratio of visceral fat to subcutaneous fat that determines metabolic abnormalities and hemodynamic consequences. This phenotype is often accompanied by an increased amount of visceral fat, but a normal or near-normal BMI and is typical for the metabolic syndrome (MS) [9]. MS was found in 20 % of hypertensive children in comparison to only 2 % of the general population [10]. Another typical metabolic abnormality in PH is the tendency for hyperuricemia; hypertensive adolescents tend to have serum uric acid concentrations in the upper range of normal values when compared to normotensive children. Feig et al. showed that elevated serum uric acid levels differentiated adolescents with PH from those with white-coat hypertension (WCH) and secondary hypertension, and finally, treatment with allopurinol lowered both uric acid levels and BP [11–13].

The presence of MS indicates exposure to excessive oxidative stress (SOX) as was shown in a few reports on SOX in hypertensive children [14–18]. In one of the first pediatric reports, Goonasekkara et al. reported that hypertensive children had significantly increased serum levels of asymmetric dimethylarginine and symmetric dimethylarginine [15]. Increased oxidative stress is typical in obese children when compared with non-obese children [16]. However, hypertensive children are exposed to greater SOX, irrespective of their BMI [17]. SOX markers also correlate with 24-h systolic BP. In a prospective study it was found that hypertensive children were exposed to greater SOX, and the SOX markers were significantly associated with left ventricular hypertrophy (LVH) and the presence of MS [18].

Metabolic abnormalities in PH are not only reversible but also determine regression of target organ damage (TOD). In a prospective study of 86 children with PH who were treated with lifestyle changes and pharmacological therapy (based on angiotensin converting enzyme inhibitors (ACEi) or angiotensin receptor blockers, ARBs), blood pressure normalized in 70 % of these patients, while TOD, SOX, and metabolic and immunological abnormalities significantly regressed. It was found that the only predictor of a decrease in the left ventricular mass index (LVMI) and the carotid wall cross-sectional area was a decrease in visceral obesity expressed by a reduction of waist circumference (WC) [19].

### Accelerated biological maturation

Other findings show that hypertensive adolescents mature at a slightly faster rate than their normotensive peers. The idea that PH is a disorder of accelerated growth was initially introduced by Lever and Harrap in 1992 [20]. They hypothesized that accelerated biological maturation is associated with metabolic alterations, including hyper-insulinism and insulin resistance (IR). Together these alterations may lead to the hypertrophy of arterial wall smooth muscle cells, along with the development of hypertension and cardiovascular disease. This hypothesis is supported by findings of Katz et al. who revealed that the bone age of hypertensive adolescents was significantly higher when compared with age-matched groups of normotensive children [21]. It was further confirmed by Cho et al., who found that sexual maturity was an independent predictor of systolic BP in both boys and girls [22]. In our study, hypertensive boys had more advanced bone age (on average by 1.5 years) when compared with normotensive peers. Moreover, advanced maturation was proportional to the level of BP; from normotension to pre-hypertension and furthermore to stage 1 and 2 hypertension [23]. Accelerated maturation is also linked with BMI and visceral obesity. A retrospective analysis from the ‘Fels Longitudinal Study’ indicated that young adults (18–35 years of age), who matured early had greater BMI, WC and cardiovascular risk factors than those who matured at a slower rate [24]. Similarly, a retrospective study from Iceland found that adult men who had their highest growth velocity between the ages of 8 and 13, had a 66 % increased risk of hypertension when compared to men who had low growth velocity [25]. The cardiovascular risk associated with increased biological maturation is not limited to males. It has been found that an earlier age at menarche correlates with higher BP, visceral obesity, and MS in adulthood [26].

### Activation of immune system in pediatric hypertension

Numerous clinical observations and experimental data indicate the role of innate and adaptive immune responses in the pathogenesis of PH. Although an innate immune system is non-specifically activated by both metabolic and hemodynamic factors, the adaptive T-cell-dependent system seems to be crucial for maintaining hypertension. This was documented by the transfer of hypertension, with lymph node cells from hypertensive to normotensive rats [27]. In the 1970s, Svendsen reported that an intact thymus was necessary for maintaining elevated BP in hypertension-prone rats and mice [28]. Importantly, the role of thymus-derived lymphocytes was not established during the early stages of the disease; however, in the chronic stage of experimental hypertension, mice with an intact thymus had more advanced degenerative changes within the arterial walls of their intra-renal arteries, when compared to nude mice with an inherited aplasia of the thymus. This led to the idea that the T-lymphocyte-dependent immune reaction is directed against antigens derived from a hemodynamically injured arterial wall [29]. These observations were further confirmed by experiments with the hypertension-prone NZB (New Zealand Black) strain of mice, in which the athymic NZB mice did not develop hypertension [30]. Since these early reports, many other studies have shown the role of intact and adaptive immune responses in the pathogenesis of arterial hypertension. It has been shown that RAG^−^/^−^ mice that do not have T-lymphocytes, do not have elevated BP during angiotensin 2 (AT2) infusions. However, the adoptive transfer of T-cells restored sensitivity to AT2 [31]. It was also found that T-regulatory cells (T-regs), which limit the extent of the immune response, play a significant role in the pathogenesis of arterial hypertension within the experimental setting. In the mouse model of arterial hypertension, an adoptive transfer of T-regs limited hypertension and organ damage induced either by AT2 or aldosterone [32].

Despite large amounts of data from experimental studies indicating the immunological basis of the pathogenesis of PH, there are relatively few reports from clinical studies involving children. Chronic sub-clinical inflammation is associated with visceral obesity, is a predictor of the development of IR and diabetes, and is considered to be a part of MS [33, 34]. A cross-sectional analysis of data obtained from children aged 8–17 years in NHANES, between 1999 and 2004, revealed that children with C-reactive protein levels above 3 mg/dl had higher systolic blood pressure than children with C-reactive protein levels below 3 mg/dl. Interestingly, that association was observed solely in boys [35]. We also found that children with PH had greater serum concentrations of highly sensitive C-reactive protein (hsCRP) and chemokines (RANTES, MIP1β), in comparison with normotensive children. The hsCRP levels correlated with MS, visceral obesity, SOX and markers of TOD, including increased carotid intima-media thickness (cIMT) and LVMI [36].

Interaction of the renin–angiotensin system (RAS) with peripheral blood leucocytes (PBL) provides another link between arterial hypertension and the immune system. RAS is active in PBL and AT2, with ARBs affecting both innate and adaptive immune systems [37–39]. In a study of gene expression in PBL in hypertensive adults, it was found that in untreated patients, the expression of 314 genes was up-regulated (including those involved in inflammatory reactions, antioxidative defense, and RAS) and 366 genes were down-regulated. Treatment led to a significant decrease of BP along with almost full normalization of the pattern of gene expression [40]. In another study in hypertensive adults, it was found that PBL provided up-regulated expression of 21 genes known to be engaged in apoptosis and inflammatory responses [41]. The interactions of the local RAS system and the immune system occur early in the course of PH; this was found in a study of children with PH observed before and after 6 months of non-pharmacological treatment. PBL from untreated children showed an increased expression of mRNA of the angiotensin-converting enzyme and CD14 genes, while the expression of angiotensinogen and AT2 type 1 receptor mRNAs were down regulated. It was also found that children with increased cIMT had a significantly lower renin gene mRNA expression [42]. There is also data indicating that psychosocial stress induces vascular dysfunction and hypertension through inflammatory mechanisms via nuclear factor kappa B which is a proinflammatory transcription factor (reviewed in [43]). It was also found that psychosocial stress mediated by beta-adrenergic receptors caused nuclear factor kappa B activation in PBL [44].

**Fig. 1:**
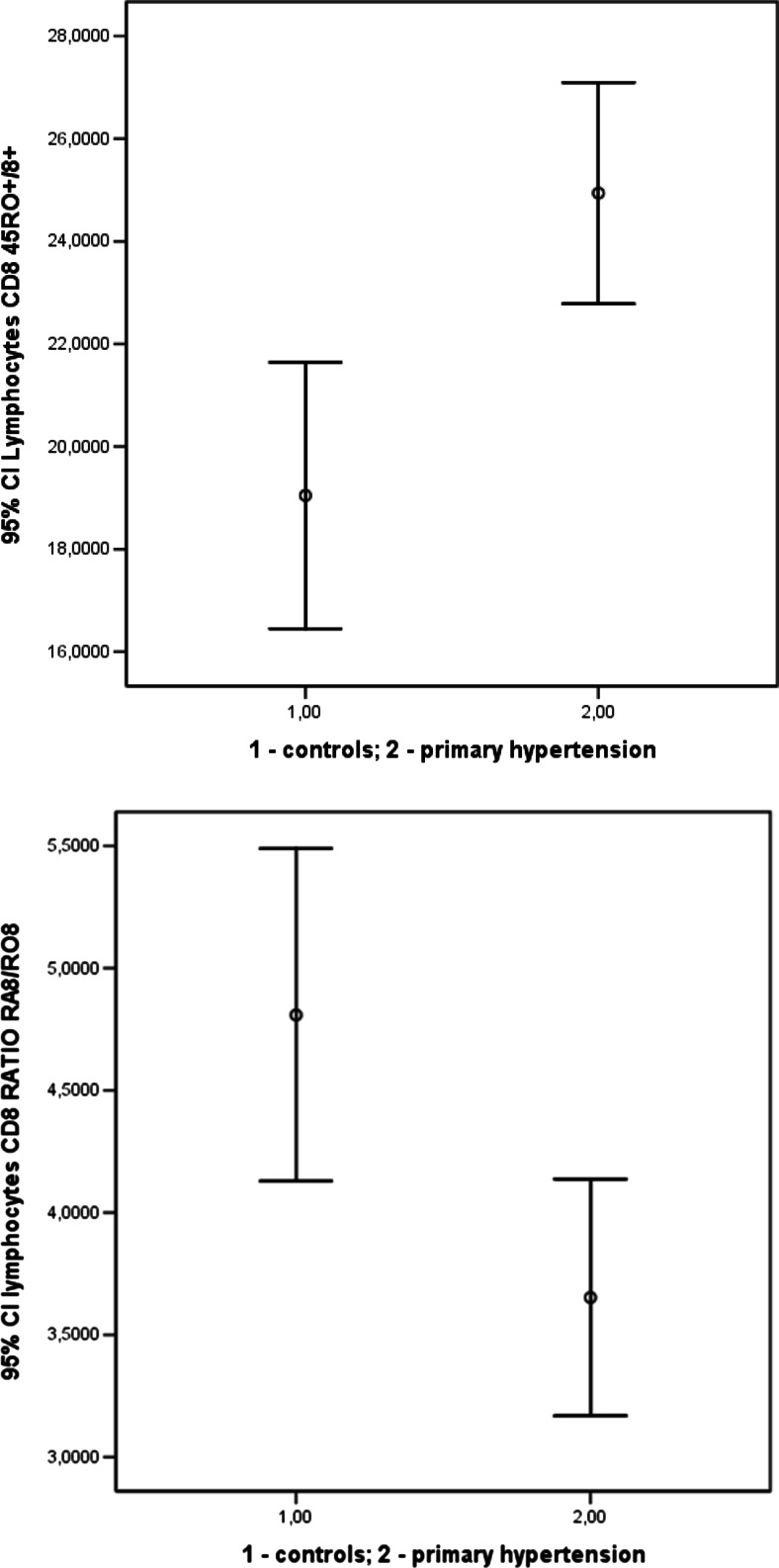
Two isoforms of common leukocyte antigen (CD45), namely CD45RO and CD45RA are expressed on “memory” and naive T cells, respectively [102]. The greater number of CD45RO-positive T cells, the greater number of memory cells. Increase in CD45RO may result from “naive” T cell activation by as yet unknown endogenous stimuli so the cells of the naive phenotype may acquire the memory phenotype (transition of RA into RO). Preliminary unpublished data: percentage of CD45RO-positive CD8 T cells (*upper panel*) and ratio of 45RA to 45RO bearing CD8 T cells (*lower panel*) in 32 normotensive, healthy adolescents vs. 72 hypertensive adolescents (both* p *< 0.05)

It has recently been reported that accelerated aging of the immune system may play a role in the pathogenesis of hypertensive arteriolosclerosis in the kidneys. It was found that adults with PH had a higher number of circulating lymphocytes with markers of aging CD57+/CD8+ and CD28-/CD8+ when compared to normotensive controls [45]. Our preliminary data indicate that untreated adolescents with PH had significantly more memory CD8^+^ T-cells than their normotensive peers; this indirectly indicates accelerated aging of the immune system (Fig. 1).

### Dysregulation of the autonomic nervous system (ANS) in children with PH

Several mechanisms leading to and maintaining central sympathetic hyperactivity in PH have been identified. Impaired vagal heart rate control exerted by an arterial baroreflex, impaired volume-sensitive cardiopulmonary reflex and arterial chemoreceptors, as well as humoral factors such as AT2, aldosterone or leptin with direct central sympatho-excitatory effects, have all been shown to play at least a partial role in hypertension (reviewed in [46]). The first reports were based on observations of elevated heart rate and cardiac output in children with elevated BP; such a hemodynamic pattern fulfilled the criteria of hyperkinetic circulation. Hemodynamic measurements in 95 adolescents, who were referred due to PH, revealed that there was a significant increase in the cardiac index from the normotensive/WCH phase, through the pre-hypertension to ambulatory and severe ambulatory hypertension phase. However, the total peripheral resistance index decreased non-significantly; this helps explain the hemodynamic mechanism in the elevation of BP [47]. In the Tecumseh Study, Julius et al. found that a child who had a faster heart rate at 7 years of age, also had higher BP values from the age of 15 to 23. Moreover, that study proved that there is a “two-way street” between obesity and PH. Children who had higher BP values at the age of 7 had a greater fat mass at the age of 22 years [48].

The relationship between obesity and increased sympathetic drive is dependent on intact pathways of signal transmission in the central nervous system, including leptin and proopiomelanocortin pathways. Obese subjects who have a mutated melanocortin receptor 4 do not present with an elevated heart rate or PH despite their severe obesity [49]. Studies of obese adults have shown that sympathetic drive measured as muscle sympathetic nerve activity (MSNA), correlates with WC. Moreover, it was found that sympathetic activation and BP elevation associated with visceral obesity depend on androgen action and are more exaggerated in boys than girls [50]. There are currently no data directly comparing the ANS activity of adolescent girls and boys, but the physiological rise in BP is observed only in boys during their pubertal growth spurt [51]. This corresponds with the dominance of boys among adolescents with PH and the ratio of boys to girls among adolescents with primary hypertension within our studies; the ratio is 3–4:1.

Another indirect method of assessment of ANS function is the analysis of cardiovascular rhythmicity. It was found that children with both WCH and PH had significantly more prevalent ultradian (12-h) cardiovascular rhythms, with reduced amplitudes and delayed acrophases in comparison to normotensive children [52]. Interestingly, a repeated analysis performed in PH patients after 12 months of treatment revealed that the abnormal pattern of cardiovascular rhythms persisted despite BP lowering. The normalization of acrophases and diurnal cardiovascular rhythms was not determined by a BP decrease, but rather by a decrease in the amount of visceral fat assessed by WC and magnetic nuclear imaging [53]. These findings suggest that sympathetic overactivity is indeed the primary disturbance in hypertensive children.

### Target organ damage and its association with metabolic and immune abnormalities

There is a continuous proportional increase of LVMI, cIMT, and arterial stiffness, related to the change in BP levels from normotension through pre-hypertension, finally to sustained hypertension [54]. In a recent meta-analysis on the relationships between BP (assessed by ambulatory BP monitoring) and TOD, it was found that hypertensive children had an LVMI value greater by 6.9 g/height^2.7^ in comparison to normotensive children [55]. Interestingly, children with WCH had an LVMI greater by 2.9 g/height^2.7^ in comparison to normotensive children [56, 57]. Hypertensive cardiac and arterial injury was found in 40 % of adolescents with PH during diagnosis, along with the presence of severe LVH in 13–15 % [58, 59]. Contrary to common belief, the most important determinant of LVH in children with PH is not BP, but rather BMI and metabolic abnormalities typical of MS. In fact, severe LVH was found solely in hypertensive adolescents who fulfilled all diagnostic criteria of MS [10]. Accordingly, Brady et al. found that BP values were relatively weak determinants of LVMI [59]. Moreover, it became apparent that the main predictor of a decreasing LVMI during antihypertensive treatment was not a decrease in BP, but rather a decrease of WC. Similarly, the decrease of cIMT has been associated with the decrease of WC and the normalization of inflammatory activity [19].

### Early aging of the cardiovascular, metabolic, immune and sympathetic system in children with PH

The above-described abnormalities associated with PH in children and adolescents, such as accelerated tempo of growth, visceral obesity with disturbed relationships between fat and muscle mass, metabolic abnormalities, increased IMT, stiffness of the arterial tree and immune alterations, are also typical markers of accelerated or premature aging [60]. These abnormalities also fit with the hypothesis of early vascular aging (EVA) as an underlying phenomenon leading to clinically overt cardiovascular disease [61]. Data from the Amsterdam Growth and Health Longitudinal Study, indicate that, individuals with stiffer carotid arteries at the age of 36 years were characterized by ages 13 to 36 years, by higher BP and steeper increases in WC, independently of each other and other risk factors. Importantly, those increases were already present in adolescence and preceded the development of dyslipidemia, a decrease of cardiorespiratory fitness and an increase in heart rate [62].

Recently, more comprehensive models of PH development that take into consideration alterations in the immune system, activation of the SNS and metabolic abnormalities, including SOX, have been proposed [63–65]. Furthermore, these models give a temporal perspective on the development of PH and its complications through the decades of life, along with a few distinct phases where different pathogenic events play a role. In the early pre-clinical stage, different stimuli send signals to the central nervous system, which initiates increased SNS activity. These signals are of different origin, and include effects of adipokines released from visceral fat, the activation of RAS with direct effects of AT2 on brain centers, metabolic abnormalities mediated by insulin and reactive oxygen species (ROS) or chronic stress. The dominant clinical manifestation in the next phase is increased sympathetic activity. TOD associated with metabolic abnormalities, SOX and hemodynamic injury to the arterial wall, are reversible during the early stages of the disease. However, with time (likely decades) and ongoing exposure to innate immune responses, the inflammatory reaction in the arterial walls of both large and small arterioles causes a release of neo-antigens [63].

The presentation of neo-antigens in dendritic cells triggers an adaptive immune response against the arterial walls with a central role being played by T-cells. This leads to irreversible changes in the arterial and arteriolar walls, including kidney vessels. The end phase consists in widespread hypertensive arterial and arteriolar remodeling, with the addition of increased arterial stiffness and development of hypertensive kidney injury. This late phase may correspond to sodium-sensitive hypertension, which is seen in the late stages of hypertensive disease.

Neo-antigens are formed by intracellular proteins; there is data indicating that neo-antigens are heat shock proteins (HSP), which are very immunogenic in an extracellular environment [66, 67]. The role of neo-antigens, and especially HSP70 along with antibodies against HSPs, has been documented in experimental models of sodium-sensitive hypertension with inflammatory changes in the kidney [67]. Studies in adults with borderline PH partially revealed elevated concentrations of HSP60 and anti-HSP65. However, in adults with established hypertension and on average older by 18 years than adults with borderline PH, antibodies against HSP70 and HSP65 were elevated, yet the levels of HSPs remained the same as the control group [68, 69]. It should be mentioned that in these studies, hypertensive patients presented with the same intermediate phenotype of overweight/obesity, visceral obesity, and insulin resistance, as was observed in the adolescent patients with PH. There are no reports on HSPs or other potential neo-antigens in children and adolescents with PH; however, studies on early atherogenesis revealed that the spectrum of elevated serum HSPs and reactivity of T-lymphocytes against different HSPs is related to age and advancement of disease. These studies showed a strict relationship between reactivity against HSP60 and cIMT in 18-year-old adolescents and revealed that reactivity of lymphocytes against HSP60 was the strongest determinant of cIMT [70, 71].

## Treatment of PH in childhood and adolescence: is rejuvenation of the arterial system possible?

Due to the fact that all main alterations in PH resemble those found in premature aging, one may say that the treatment should focus on rejuvenation of the vascular system. It was found that immunosuppressive treatment directed against T-lymphocytes, or applying mycophenolate mofetil, were both effective in animal models of hypertension [72]. It was also found that patients with rheumatoid arthritis treated with mycophenolate mofetil, had a lower prevalence of arterial hypertension when compared to other patients [73]. However, due to important severe side effects, immunosuppressive treatment in the form of immunosuppressive drugs does not seem to be a practical solution for the treatment of PH. There were several attempts to use antioxidants as a solitary or supplementary therapy in adults with PH, but the results of those trials have remained disappointing [74]. Thus, there remain two main forms of antihypertensive treatment; non-pharmacological and pharmacological therapy.

Non-pharmacological treatment is based on daily physical activity and diet. According to the guidelines, it is recommended to start pharmacological treatment in all cases of life-threatening and secondary hypertension. In cases of PH in children, pharmacological treatment should commence with TOD and/or with stage 2 hypertension or when non-pharmacological treatment is ineffective. Most children and adolescents with PH present with stage 1 hypertension with only subtle TOD and should therefore begin non-pharmacological therapy. The efficiency of non-pharmacological treatment based on vigorous physical exercise and diet depends on the motivation and conscious decisions made by the patient in reference to make a change to his/her lifestyle. The effects are quite small in adults but quite encouraging in children and adolescents. The treatment should be focused towards decreasing visceral fat and increasing fitness level; physical exercise exerts significant positive effects, which act at a molecular level on the main disturbances found in PH [75]. Preventive and therapeutic effects of structured exercise training during adulthood, is well documented [76]. Importantly, physical fitness exerts a strong modifying effect on BP trajectories over the life span of an adult male. Results from the Aerobics Centre Longitudinal Study, including 13,953 men between 20 and 90 years of age without hypertension, cardiovascular disease or cancer, who completed 3 to 28 (mean of 3.8) follow-up medical examinations between 1970 and 2006, showed that subjects with higher fitness levels experienced an SBP increase later than those with low fitness levels [77]. Interventional studies in children showed that physical activity is associated with the lowering of BP in 11–12-year-old obese and hypertensive children. It was also found that volume is more important than intensity when referring to physical activity [78]. In one of the first reports on the effects of non-pharmacological therapies on obese children, it was found that both exercise and diet caused significant hypotensive effects and increased post-ischemic dilation of the forearm vessels. However, the effects of exercise programs were significantly greater in terms of BP reduction, improvement in cardiovascular fitness and endothelial function when compared to diet alone [79]. Similarly, Woo et al. documented that diet plus exercise caused greater positive effects on endothelial function in 9–12-year-old obese children when compared to diet alone. Significant improvement of flow-mediated dilation in the brachial artery was evident after only 6 weeks, with sustained improvement being observed in those who exercised regularly for 1 year [80].

When deciding on pharmacological treatment, one may choose from eight different groups of antihypertensive medications. However, some of these medications may also cause more harm than benefit when used in adolescents with PH; this is not considered BP lowering, but rather a decrease in cardiovascular risk.

Thiazides are the most commonly prescribed antihypertensive drugs worldwide; however, their use poses the risk of DM and worsening of metabolic abnormalities [81]. A recent meta-analysis of 22 clinical trials with 143,153 non-DM patients revealed that antihypertensive treatment, with a combination of diuretics, was associated with an increased risk of new-onset DM when compared to other antihypertensive agents or placebo [82]. Also, the ALLHAT study showed that chlorthalidone produced a greater increase in the fasting glucose level and a significantly higher risk of DM than that of amlodipine or lisinopril [83]. Therefore, thiazides and other diuretics should not be used as the first or even second-line drug of choice, regarding the treatment of PH in children and adolescents.

Although beta-blockers slow down the heart rate and decrease BP, their primary use is associated with the development of obesity and IR [84]. Weight gain during beta-blocker therapy is mainly related to reduced energy expenditure. Sharma et al. showed that beta-blockers in obese hypertensive patients reduced the basal metabolic rate by 12 % when compared with other antihypertensive therapies [85]. Furthermore, beta-blockers decreased insulin secretion from pancreatic β-cells along with blood flow in the skeletal muscles, which led to the impairment of glucose metabolism and IR. A large meta-analysis of hypertensive patients treated with beta-blockers revealed an increased risk of new-onset DM compared to other non-diuretic antihypertensive agents [86]. However, it seems that there are significant differences throughout the spectrum of adverse metabolic effects caused by non-selective and selective beta-blockers. It was found that a long-acting form of metoprolol, which is a selective beta1-blocker, did not aggravate insulin resistance [87, 88]. Nevertheless, the direct comparison of nebivolol and metoprolol indicated that nebivolol had a better metabolic profile as it did not increase IR or SOX [89, 90]. In view of these potentially severe adverse effects, beta-blockade should not be used as first-line therapy in children and adolescents. However, new beta-blockers such as nebivolol, are devoid of the negative effects of old beta-blockers and may be an alternative for adolescent girls with PH, in whom the use of blockers of the RAS system can be problematic due to the adverse effects of RAS blockers in pregnancy.

From a pathophysiological point of view, the best choices in pharmacological therapy of PH are drugs which exert not only hypotensive effects, but also positive metabolic effects. The best choices are dihydropyridine calcium channel blockers (CCBs) which are metabolically neutral and blockers of the renin–angiotensin system – ACE inhibitors (ACEi) and AT2 receptor blockers (ARBs).

CCBs are generally considered as having a low potential to impair the metabolic profile. Indeed, a meta-analysis of ten randomized clinical trials evaluated the effects of antihypertensive drugs on glucose metabolism. It was found that the risk of new-onset DM among subjects taking CCBs was not significantly greater compared to patients treated with beta-blockers or diuretics. However, CCBs were associated with a higher incidence of DM than ACEi [91]. On the other hand, a recent re-analysis of data from the NAVIGATOR trial showed that CCBs were not associated with a higher risk of new-onset DM [92].

The majority of clinical trials evaluating the effects of ACEi and ARBs on glucose homeostasis have revealed that inhibition of the RAS system was associated with increased insulin sensitivity, better glucose uptake by the skeletal muscles and a lower incidence of new-onset DM in hypertensive subjects. The HOPE study demonstrated a favorable influence of ramipril on cardiovascular incidents in high-risk patients and a reduction of new-onset DM by 34 % when compared to placebo [93]. The ALLHAT trial, which evaluated the influence of CCBs, ACEi and diuretics on cardiovascular events, revealed that lisinopril reduced the relative risk of developing DM by 30 % when compared to chlorthalidone and by 17 % when compared to amlodipine therapy [83]. In addition, the LIFE study, which enrolled 9,193 patients treated with atenolol or losartan, showed a significantly reduced new-onset DM in the losartan group compared to the atenolol group [94]. Similar results have also been presented in other studies [95, 96]. Some findings have indicated that weight loss in obese individuals was associated with the inhibition of RAS [97]. This may be partly due to reduced SNS activity, resulting in decreased renin release and change in adipocyte function. The inhibition of RAS may result in a shift in adipocyte distribution from visceral to subcutaneous depots. Furthermore, there is a correlation between 24-h BP and the expression of genes related to RAS in adipocytes [98, 99]. Moreover, it has been suggested that AT2 increases SOX in human pre-adipocytes and can be inhibited by ARB. Telmisartan was more effective in this field than other ARBs. It was found that telmisartan improved adiponectin secretion in pre-adipocytes and had beneficial metabolic effects via selective peroxisome proliferator-activated receptor-γ modulation [100]. In addition, patients treated with ACEi or ARBs showed a significant reduction in visceral fat, compared to other antihypertensive drugs. In a study involving 54 Japanese patients with MS and abdominal obesity, telmisartan was associated with a significant reduction in the plasma glucose/insulin levels and visceral fat tissue, whereas amlodipine had no effect [101].

## Conclusions

We have concluded that primary hypertension in children and adolescents is not a purely hemodynamic phenomenon, but rather an immune-metabolic syndrome associated with an increased sympathetic drive. The main intermediate phenotypes of PH in children resemble those observed during normal aging. The best initial treatment of PH is the combination of non-pharmacological therapy based on physical exercise and diet. In some patients, additional use of antihypertensive medications, ideally blockers of RAS are needed. These can lead to rejuvenation of the vascular system and improvement of metabolic abnormalities.

## References

[1] Page IH (1949). Pathogenesis of arterial hypertension. J Am Med Assoc.

[2] Harrison DG (2013). The mosaic theory revisited: common molecular mechanisms coordinating diverse organ and cellular events in hypertension. J Am Soc Hypertens.

[3] Paradis G, Lambert M, O’Loughlin J, Lavallée C, Aubin J, Delvin E, Lévy E, Hanley JA (2004). Blood pressure and adiposity in children and adolescents. Circulation.

[4] Sorof J, Daniels S (2002). Obesity hypertension in children: a problem of epidemic proportions. Hypertension.

[5] Flynn JT, Alderman MH (2005). Characteristics of children with primary hypertension seen at a referral centre. Pediatr Nephrol.

[6] Litwin M, Trelewicz J, Wawer Z, Antoniewicz J, Wierzbicka A, Rajszys P, Grenda R (2004). Intima-media thickness and arterial elasticity in hypertensive children: controlled study. Pediatr Nephrol.

[7] Srinivasan SR, Myers L, Berenson GS (2006). Changes in metabolic syndrome variables since childhood in pre-hypertensive and hypertensive subjects: the Bogalusa Heart Study. Hypertension.

[8] Sinaiko A, Steinberger J, Moran A, Hong C, Prineas R, Jacobs D (2006). Influence of insulin resistance and body mass index at Age 13 on systolic blood pressure, triglycerides, and high-density lipoprotein cholesterol at Age 19. Hypertension.

[9] Pludowski P, Litwin M, Sladowska J, Antoniewicz J, Niemirska A, Wierzbicka A, Lorenc RS (2008). Bone mass and body composition in children and adolescents with primary hypertension: preliminary data. Hypertension.

[10] Litwin M, Sladowska J, Antoniewicz J, Niemirska A, Wierzbicka A, Daszkowska J, Wawer ZT, Janas R, Grenda R (2007). Metabolic abnormalities, insulin resistance, and metabolic syndrome in children with primary hypertension. Am J Hypertens.

[11] Feig DI, Johnson RJ (2003). Hyperuricemia in childhood primary hypertension. Hypertension.

[12] Feig DI, Johnson RJ (2007). The role of uric acid in pediatric hypertension. J Ren Nutr.

[13] Feig DI, Soletsky B, Johnson RJ (2008). Effect of allopurinol on blood pressure of adolescents with newly diagnosed essential hypertension: a randomized trial. JAMA.

[14] Redón J, Oliva MR, Tormos C, Giner V, Chaves J, Iradi A, Sáez GT (2003). Antioxidant activities and oxidative stress byproducts in human hypertension. Hypertension.

[15] Goonasekera CD, Rees DD, Woolard P, Frend A, Shah V, Dillon MJ (1997). Nitric oxide synthase inhibitors and hypertension in children and adolescents. J Hypertens.

[16] Ostrow V, Wu S, Aguilar A, Bonner R, Suarez E, De Luca F (2011). Association between oxidative stress and masked hypertension in a multi-ethnic population of obese children and adolescents. J Pediatr.

[17] Túri S, Friedman A, Bereczki C, Papp F, Kovàcs J, Karg E, Németh I (2003). Oxidative stress in juvenile essential hypertension. J Hypertens.

[18] Sladowska-Kozłowska J, Litwin M, Niemirska A, Płudowski P, Wierzbicka A, Skorupa E, Wawer ZT, Janas R (2012). Oxidative stress in hypertensive children before and after 1 year of antihypertensive therapy. Pediatr Nephrol.

[19] Litwin M, Niemirska Ś-KJ, Wierzbicka A, Janas R, Wawer ZT, Wisniewski A, Feber J (2010). Regression of target organ damage in children and adolescents with primary hypertension. Pediatr Nephrol.

[20] Lever AF, Harrap SB (1992). Essential hypertension: a disorder of growth with origins in childhood?. J Hypertens.

[21] Katz SH, Hediger ML, Schall JI, Bowers IJ, Barker WF, Aurand S, Eveleth PB, Gruskin AB, Parks JS (1980). Blood pressure, growth and maturation from childhood through adolescence: mixed longitudinal analyses from Philadelphia Blood Pressure Project. Hypertension.

[22] Cho SD, Mueller W, Meininger JC, Liehr P, Chan W (2001). Blood pressure and sexual maturity In adolescents: the Heartfelt Study. Am J Hum Biol.

[23] Pludowski P, Litwin M, Niemirska A, Jaworski M, Sladowska J, Kryskiewicz E, Karczmarewicz E, Neuhoff-Murawska J, Wierzbicka A, Lorenc RS (2009). Accelerated skeletal maturation in children with primary hypertension. Hypertension.

[24] Sun SS, Schubert CM (2009). Prolonged juvenile states and delay of cardiovascular and metabolic risk factors: The Fels Longitudinal Study. J Pediatr.

[25] Halldorsson T, Gunnarsdottir I, Birgisdottir BE, Gudnason V, Aspelund T, Thorsdottir I (2010). Childhood growth and adult hypertension in a population of high birth weight. Hypertension.

[26] Kivimaki M (2008). Association of age at menarche with cardiovascular risk factors, vascular structure, and function in adulthood: The Cardiovascular Risk in Young Finns Study. Am J Clin Nutr.

[27] Okuda T, Grollman (1967). Passive transfer of autoimmune induced hypertension in the rat by lymph node cells. Tex Rep Biol Med.

[28] Svendsen UG (1975). Studies elucidating the importance of thymus on the degree of increased blood pressure and vascular disease in renal hypertensive mice. A comparison of the disease in nude and haired littermates. Acta Pathol Microbiol Scand A.

[29] Svendsen UG (1976). The role of thymus for the development and prognosis of hypertension and hypertensive vascular disease in mice following renal infarction. Acta Pathol Microbiol Scand A.

[30] Svendsen UG (1977). Spontaneous hypertension and hypertensive vascular disease in the NZB strain of mice. Acta Pathol Microbiol Scand A.

[31] Guzik TJ, Hoch NE, Brown KA, McCann LA, Rahman A, Dikalov S, Goronzy J, Weyand C, Harrison DG (2007). Role of the T cell in the genesis of angiotensin II induced hypertension and vascular dysfunction. J Exp Med.

[32] Barhoumi T, Kasal DA, Li MW, Shbat L, Laurant P, Neves MF, Paradis P, Schiffrin EL (2011). T regulatory lymphocytes prevent angiotensin II-induced hypertension and vascular injury. Hypertension.

[33] Chae CU, Lee RT, Rifai N, Ridker PM (2001). Blood pressure and inflammation in apparently healthy man. Hypertension.

[34] Festa A, Jr A, Howard G, Mykkänen L, Tracy RP, Haffner SM (2000). Chronic subclinical inflammation as a part of the insulin resistance syndrome: the Insulin Resistance Atherosclerosis Study (IRAS). Circulation.

[35] Lande MB, Pearson TA, Vermilion RP, Auinger P, Fernandez ID (2008). Elevated blood pressure, race/ethnicity, and C-reactive protein levels in children and adolescents. Pediatrics.

[36] Litwin M, Michalkiewicz J, Niemirska A, Gackowska L, Kubiszewska I, Wierzbicka A, Wawer ZT, Janas R (2010). Inflammatory activation in children with primary hypertension. Pediatr Nephrol.

[37] Jurewicz M, McDermott DH, Sechler JM, Tinckam K, Takakura A, Carpenter CB, Milford E, Abdi R (2007). Human T and natural killer cells possess a functional renin–angiotensin system: further mechanisms of angiotensin-induced inflammation. J Am Soc Nephrol.

[38] Hoch NE, Guzik TJ, Chen W, Deans T, Maalouf SA, Gratze P, Weyand C, Harrison DG (2009). Regulation of T cell function by endogenously produced angiotensin II. Am J Physiol Regul Integr Comp Physiol.

[39] Benicky J, Sanchez-Lemus E, Pavel J, Saavedra JM (2009). Anti-inflammatory effects of angiotensin receptor blockers in the brain and the periphery. Cell Moll Neurobiol.

[40] Chon H, Gaillard CAJM, van der Meijden BB, Dijstelbloem HM, Kraaijenhagen RJ, van Leenen D, Holstege FC, Joles JA, Bluyssen HA, Koomans HA, Braam B (2004). Broadly altered gene expression in blood leukocytes in essential hypertension is absent during treatment. Hypertension.

[41] Coppo M, Bandinelli M, Berni A, Galastri S, Abbate R, Poggesi L, Marra F, Gensini GF, Boddi M (2011). Ang-II upregulation of T lymphocyte renin–angiotensin system is amplified by low-grade inflammation in human hypertension. Am J Hypertens.

[42] Litwin M, Michałkiewicz TJ, Niemirska A, Wierzbicka A, Szalecki M (2013). Altered genes profile of renin–angiotensin system, immune system, and adipokines receptors in leukocytes of children with primary hypertension. Hypertension.

[43] Bierhaus A, Humpert PM, Nawroth PP (2004). NF-kappaB as a molecular link between psychosocial stress and organ dysfunction. Pediatr Nephrol.

[44] Bierhaus A, Wolf J, Andrassy M, Rohleder N, Humpert PM, Petrov D, Ferstl R, von Eynatten M, Wendt T, Rudofsky G, Joswig M, Morcos M, Schwaninger M, McEwen B, Kirschbaum C, Nawroth PP (2003). A mechanism converting psychosocial stress into mononuclear cell activation. Proc Natl Acad Sci U S A.

[45] Youn J-C, Yu HT, Lim BJ, Koh MJ, Lee J, Chang D-Y, Choi YS, Lee S-H, Kang S-M, Jang Y, Yoo OJ, Shin E-C, Park S (2013). Immunosenescent CD8+ T cells and C-X-C chemokine receptor type 3 chemokines are increased in human hypertension. Hypertension.

[46] Feber J, Ruzicka M, Geier P, Litwin M (2014). Autonomic nervous system dysregulation in pediatric hypertension. Curr Hypertens Rep.

[47] Niemirska A, Obrycki Ł, Wojciechowska E, Litwin M (2014). Hemodynamics of primary hypertension in children. Pediatr Nephrol.

[48] Julius S, Valentini M, Palatini P (2000). Overweight and Hypertension: a two-way street?. Hypertension.

[49] Greenfield JR, Miller JW, Keogh JM, Henning E, Satterwhite JH, Cameron GS, Astruc B, Mayer JP, Brage S, See TC, Lomas DJ, O’Rahilly S, Farooqi IS (2009). Modulation of blood pressure by central melanocortinergic pathways. N Engl J Med.

[50] Syme C, Abrahamowicz M, Leonard GT, Perron M, Pitiot A, Qiu X, Richer L, Totman J, Veillette S, Xiao Y, Gaudet D, Paus T, Pausova Z (2008). Intra-abdominal adiposity and individual components of the metabolic syndrome in adolescence: sex differences and underlying mechanisms. Arch Pediatr Adolesc Med.

[51] Kułaga Z, Litwin M, Grajda A, Kułaga K, Gurzkowska B, Góźdź M, Pan H; OLAF Study Group (2012). Oscillometric blood pressure percentiles for Polish normal weight school-aged children and adolescents. J Hypertens.

[52] Litwin M, Simonetti GD, Niemirska A, Ruzicka M, Wühl E, Schaefer F, Feber J (2010). Altered cardiovascular rhythmicity in children with white coat and ambulatory hypertension. Pediatr Res.

[53] Niemirska A, Litwin M, Feber J, Jurkiewicz E (2013). Blood pressure rhythmicity and visceral Fat in children with hypertension. Hypertension.

[54] Stabouli S, Kotsis V, Rizos Z, Toumanidis S, Karagianni C, Constantopoulos A, Zakopoulos N (2009). Left ventricular mass in normotensive, pre-hypertensive and hypertensive children and adolescents. Pediatr Nephrol.

[55] Kollias A, Dafni M, Poulidakis E, Ntineri A, Stergiou GS (2014). Out of office blood pressure and target organ damage in children and adolescents: a systematic review and meta-analysis. J Hypertens.

[56] Litwin M, Niemirska A, Ruzicka M, Feber J (2009). White coat hypertension in children: not rare and not benign?. J Am Soc Hypertens.

[57] Sorof JM, Cardwell G, Franco K, Portman RJ (2002). Ambulatory blood pressure and left ventricular mass in hypertensive children. Hypertension.

[58] Ltwin M, Niemirska A, Sladowska J, Antoniewicz J, Daszkowska J, Wierzbicka A, Wawer ZT, Grenda R (2006). Left ventricular hypertrophy and arterial wall thickening in children with essential hypertension. Pediatr Nephrol.

[59] Brady TM, Fivush B, Flynn JT, Parekh R (2008). Ability of blood pressure to predict left ventricular hypertrophy in children with primary hypertension. J Pediatr.

[60] Gerhard-Herman M, Smoot LB, Wake N, Kieran MW, Kleinman ME, Miller DT, Schwartzman A, Giobbie-Hurder A, Neuberg D, Gordon LB (2012). Mechanisms of premature vascular aging in children with Hutchinson–Gilford progeria syndrome. Hypertension.

[61] Kotsis V, Stabouli S, Karafillis I, Nilsson P (2011). Early vascular aging and the role of central blood pressure. J Hypertens.

[62] Ferreira I, van de Laar RJ, Prins MH, Twisk JW, Stehouwer CD (2012). Carotid stiffness in young adults: a life-course analysis of its early determinants: the Amsterdam Growth and Health Study. Hypertension.

[63] Harrison DG1, Guzik TJ, Lob HE, Madhur MS, Marvar PJ, Thabet SR, Vinh A, Weyand CM (2011). Inflammation, immunity, and hypertension. Hypertension.

[64] Rodríguez-Iturbe B, Pons H, Quiroz Y, Johnson RJ (2014). The immunological basis of hypertension. Am J Hypertens.

[65] Rodriguez-Iturbe B, Vaziri ND, Herrera-Acosta J, Johnson RJ (2004). Oxidative Stress, Renal infiltration of immune cells and salt-sensitive hypertension: all for one and one for all. Am J Physiol Renal Physiol.

[66] Parra G, Quiroz Y, Salazar J, Bravo Y, Pons H, Chavez M, Rj J, Rodriguez-Iturbe B (2008). Experimental induction of salt-sensitive hypertension is associated with lymphocyte proliferative response to HSP70. Kidney Int.

[67] Pons H, Ferrebuz A, Quiroz Y, Romero-Vasquez F, Parra G, Johnson RJ, Rodriguez-Iturbe B (2013). Immune reactivity to heat shock protein 70 expressed in the kidney is cause of salt sensitive hypertension. Am J Physiol Renal Physiol.

[68] Frostegard J, Lemne C, Andersson B, van der Zee R, Kiessling R, deFaire U (1997). Association of serum antibodies to heat-shock protein 65 with borderline hypertension. Hypertension.

[69] Pockley AG, Wu R, Lemne C, Kiessling R, de Faire U, Frostegard J (2000). Circulating heat shock protein 60 is associated with early cardiovascular Disease. Hypertension.

[70] Knoflach M, Bernhard D, Wick G (2005). Anti-HSP60 immunity is already associated with atherosclerosis early in life. Ann N Y Acad Sci.

[71] Knoflach M, Kiechl S, Mayrl B, Kind M, Gaston JS, van der Zee R, Faggionato A, Mayr A, Willeit J, Wick G (2007). T-cell reactivity against HSP60 relates to early but not advanced atherosclerosis. Atherosclerosis.

[72] Bravo Y, Quiroz Y, Ferrebuz A, Vaziri ND, Rodríguez-Iturbe B (2007). Mycophenolate mofetil administration reduces renal inflammation, oxidative stress, and arterial pressure in rats with lead-induced hypertension. Am J Physiol Renal Physiol.

[73] Herrera J, Ferrebuz A, MacGregor EG, Rodriguez-Iturbe B (2006). Mycophenolate mofetil treatment improves hypertension in patients with psoriasis and rheumatoid arthritis. J Am Soc Nephrol.

[74] Baradaran A, Nasri H, Rafieian-Kopaei M (2014). Oxidative stress and hypertension: Possibility of hypertension therapy with antioxidants. J Res Med.

[75] Roque FR, Hernanz R, Salaices M, Briones AM (2013). Exercise training and cardiometabolic diseases: focus on the vascular system. Curr Hypertens Rep.

[76] Kokkinos P (2014). Cardiorespiratory fitness, exercise, and blood pressure. Hypertension.

[77] Liu J, Sui X, Lavie CJ, Zhou H, Park YM, Cai B, Liu J, Blair SN (2014). Effects of cardiorespiratory fitness on blood pressure trajectory with aging in a cohort of healthy men. J Am Coll Cardiol.

[78] Leary SD, Ness AR, Smith GD, Cs M, Deere K, Blair SN, Riddoch C (2008). Physical activity and blood pressure in childhood: findings from a population based study. Hypertension.

[79] Rocchini AP, Katch V, Anderson J, Hinderliter J, Becque J, Martin M, Marks C (1988). Blood pressure in obese adolescents: effect of weight loss. Pediatrics.

[80] Woo KS, Chook P, Yu CW, Sung RY, Qiao M, Leung SS, Lam CW, Metreweli C, Celermajer DS (2004). Effects of diet and exercise on obesity-related vascular dysfunction in children. Circulation.

[81] Messerli FH, Bangalore S, Julius S (2008). Risk/benefit assessment of beta-blockers and diuretics precludes their use for first-line therapy in hypertension. Circulation.

[82] Elliott WJ, Meyer PM (2007). Incident diabetes in clinical trials of antihypertensive drugs: a network meta-analysis. Lancet.

[83] Barzilay JI, Davis BR, Cutler JA, Pressel SL, Whelton PK, Basile J, Margolis KL, Ong ST, Sadler LS, Summerson J; ALLHAT Collaborative Research Group (2006). Fasting glucose levels and incident diabetes mellitus in older non-diabetic adults randomized to receive 3 different classes of antihypertensive treatment: a report from the Antihypertensive and Lipid-Lowering Treatment to Prevent Heart Attack Trial (ALLHAT). Arch Intern Med.

[84] Rizos CV, Elisaf MS (2014). Antihypertensive drugs and glucose metabolism. World J Cardiol.

[85] Sharma AM, Pischon T, Hardt S, Kunz I, Luft FC (2001). Hypothesis: Beta-adrenergic receptor blockers and weight gain: a systematic analysis. Hypertension.

[86] Bangalore S, Parkar S, Grossman E, Messerli FH (2007). A meta-analysis of 94,492 patients with hypertension treated with beta blockers to determine the risk of new-onset diabetes mellitus. Am J Cardiol.

[87] Falkner B, Francos G, Kushner H (2006). Metoprolol succinate, a selective b-adrenergic blocker, has no effect on insulin sensitivity. J Clin Hypertens (Greenwich).

[88] Falkner B, Kushner H (2008). Treatment with metoprolol succinate, a selective beta adrenergic blocker, lowers blood pressure without altering insulin sensitivity in diabetic patients. J Clin Hypertens (Greenwich).

[89] Agabiti Rosei E, Rizzoni D (2007). Metabolic profile of nebivolol, a beta-adrenoceptor antagonist with unique characteristics. Drugs.

[90] Ayers K, Byrne LM, DeMatteo A, Brown NJ (2012). Differential effects of nebivolol and metoprolol on insulin sensitivity and plasminogen activator inhibitor in the metabolic syndrome. Hypertension.

[91] Noto H, Goto A, Tsjimoto T, Noda M (2013). Effect of calcium channel blockers on incidence of diabetes: a meta-analysis. Diabetes Metab Syndr Obes.

[92] Shen L, Shah BR, Reyes EM, Thomas L, Wojdyla D, Diem P, Leiter LA, Charbonnel B, Mareev V, Horton ES, Haffner SM, Soska V, Holman R, Bethel MA, Schaper F, Sun JL, McMurray JJ, Califf RM, Krum H (2013). Role of diuretics, β blockers, and statins in increasing the risk of diabetes in patients with impaired glucose tolerance: reanalysis of data from the NAVIGATOR study. BMJ.

[93] Yusuf S, Sleight P, Pogue J, Bosch J, Davies R, Dagenais G (2000). Effects of an angiotensin-converting-enzyme inhibitor, ramipril, on cardiovascular events in high-risk patients. The Heart Outcomes Prevention Evaluation Study Investigators. N Engl J Med.

[94] Dahlöf B, Devereux RB, Kjeldsen SE, Julius S, Beevers G, de Faire U, Fyhrquist F, Ibsen H, Kristiansson K, Lederballe-Pedersen O, Lindholm LH, Nieminen MS, Omvik P, Oparil S, Wedel H, LIFE Study Group (2002). Cardiovascular morbidity and mortality in the Losartan Intervention For Endpoint reduction in hypertension study (LIFE): a randomized trial against atenolol. Lancet.

[95] Hansson L, Lindholm LH, Niskanen L, Lanke J, Hedner T, Niklason A, Luomanmäki K, Dahlöf B, de Faire U, Mörlin C, Karlberg BE, Wester PO, Björck JE (1999). Effect of angiotensin-converting-enzyme inhibition compared with conventional therapy on cardiovascular morbidity and mortality in hypertension: the Captopril Prevention Project (CAPPP) randomized trial. Lancet.

[96] Abuissa H, Jones PG, Marso SP, O’Keefe JH (2005). Angiotensin-converting enzyme inhibitors or angiotensin receptor blockers for prevention of type 2 diabetes: a meta-analysis of randomized clinical trials. J Am Coll Cardiol.

[97] Engeli S, Böhnke J, Gorzelniak K, Janke J, Schling P, Bader M, Luft FC, Sharma AM (2005). Weight loss and the renin-angiotensin-aldosterone system. Hypertension.

[98] Dharma AM (2002). Adipose tissue: a mediator of cardiovascular risk. Int J Obes Relat Metab Disord.

[99] Gorzelniak K, Engeli S, Janke J, Luft FC, Sharma AM (2002). Hormonal regulation of the human adipose-tissue renin–angiotensin system: relationship to obesity and hypertension. J Hypertens.

[100] Benson SC, Pershadsingh HA, Ho CI, Chittiboyina A, Desai P, Pravenec M, Qi N, Wang J, Avery MA, Kurtz TW (2004). Identification of telmisartan as a unique angiotensin II receptor antagonist with selective PPARgamma-modulating activity. Hypertension.

[101] Shimabukuro M, Tanaka H, Shimabukuro T (2007). Effects of telmisartan on fat distribution in individuals with the metabolic syndrome. J Hypertens.

[102] Michalkiewicz J, Barth C, Chrzanowska K, Gregorek H, Syczewska M, Weemeas CMB, Madaliński K, Dzierżanowska D, Stachowski J (2003). Abnormalities in the T and NK lymphocyte phenotype in patients with Nijmegen breakage syndrome. Clin Exp Immunol.

